# Whole Genome Resequencing Revealed the Genetic Relationship and Selected Regions among Baicheng-You, Beijing-You, and European-Origin Broilers

**DOI:** 10.3390/biology12111397

**Published:** 2023-11-03

**Authors:** Kai Yang, Jian Zhang, Yuelei Zhao, Yonggang Shao, Manjun Zhai, Huagui Liu, Lifan Zhang

**Affiliations:** 1 College of Animal Science and Technology, Nanjing Agricultural University, Nanjing 210095, China; kaiyang163njau@163.com (K.Y.); 2023205005@stu.njau.edu.cn (Y.Z.); 2 Institute of Animal Husbandry and Veterinary Medicine, Beijing Academy of Agriculture and Forestry Sciences, Beijing 100097, China; zjcau@126.com (J.Z.); liuhuagui66@163.com (H.L.); 3 College of Animal Science, Xinjiang Agricultural University, Urumqi 830052, China; ygshao@yeah.net (Y.S.); zhaimanjun@yeah.net (M.Z.)

**Keywords:** genome characteristic, Baicheng-You, Beijing-You, broiler, resequencing

## Abstract

**Simple Summary:**

Most Chinese local breeds have slow growth speed and high fat deposition, while European commercial chicken strains belong to fast-growing chicken breeds with low fat deposition. In general, the word “You” in China represents excessive fat deposition, and You-chicken is considered to be a typical fatten broiler. Among more than 100 local chickens in China, Baicheng-You and Beijing-You chickens are only two kinds of You-chicken breeds. However, their special genomic characteristics have not been well studied. This study analyzed the genome changes among these chicken breeds and obtained many candidate genes related to important traits of chickens, e.g., fat deposition, immune response, and melanin production. Our data provide valuable insights for understanding the formation of germplasm characteristics of these broiler breeds, which can be used to better protect and utilize the Chinese-You chickens.

**Abstract:**

As the only two You-chicken breeds in China, Baicheng-You (BCY) and Beijing-You (BJY) chickens are famous for their good meat quality. However, so far, the molecular basis of germplasm of the two You-chicken breeds is not yet clear. The genetic relationship among BCY, BJY, and European-origin broilers (BRs) was analyzed using whole genome resequencing data to contribute to this issue. A total of 18,852,372 single nucleotide polymorphisms (SNPs) were obtained in this study. After quality control, 8,207,242 SNPs were applied to subsequent analysis. The data indicated that BJY chickens possessed distant distance with BRs (genetic differentiation coefficient (*F*_ST_) = 0.1681) and BCY (*F*_ST_ = 0.1231), respectively, while BCY and BRs had a closer relationship (*F*_ST_ = 0.0946). In addition, by using *F*_ST_, cross-population extended haplotype homozygosity (XP-EHH), and cross-population composite likelihood ratio (XP-CLR) methods, we found 374 selected genes between BJY and BRs chickens and 279 selected genes between BCY and BJY chickens, respectively, which contained a number of important candidates or genetic variations associated with feather growth and fat deposition of BJY chickens and potential disease resistance of BCY chickens. Our study demonstrates a genome-wide view of genetic diversity and differentiation among BCY, BJY, and BRs. These results may provide useful information on a molecular basis related to the special characteristics of these broiler breeds, thus enabling us to better understand the formation mechanism of Chinese-You chickens.

## 1. Introduction

In the list of chicken genetic resources in China, only two indigenous You-chicken breeds, namely Baicheng-You (BCY) and Beijing-You (BJY), are well known for their fat characteristics [1]. In general, the Chinese word “You” often means excessive fat deposition, which is considered a crucial economic trait in the broiler industry because of its correlation with meat quality and the cost of production. Among these two chicken breeds, BJY chickens originate from Beijing and have yellow feathers, tender meat, high fat deposition, and a unique appearance, such as feathered legs and polydactyly [1,2]. BCY chickens originate from Baicheng, Xinjiang province, which is nearly 4000 km away from Beijing. BCY chickens are mainly black and brown, show strong resistance to disease and cold, and get their name from the presence of a fat layer under the skin after fattening [1,3]. Currently, with the development of the economy, there is an increasing demand for high-quality animal products. As famous local chicken breeds in China, BCY and BJY chickens can provide fresh and delicious chicken products and be used as breeding materials to cultivate new synthetic lines or breeds with high meat quality, indicating that these two chicken breeds have good market development prospects. However, to date, the genetic discrepancy between the two Chinese-You chickens remains unknown.

In recent years, as a new sequencing technique that can detect all genomic information of individuals, genome resequencing has become the primary method for studying population genetics in chickens. For example, discovering signatures of selection in Iranian indigenous chicken ecotypes [4], digging the genomic adaptive changes in modern chickens [5], identifying the genes associated with tropical and frigid environments in chickens [6], skin color in Xichuan black-bone chickens [7], and the genetic basis of meat yield in chickens [8]. These data demonstrate that genome resequencing is a comprehensive and reliable method to explore selected regions and important functional genes related to economically important traits in chickens.

However, knowledge of the germplasm characteristics of these two Chinese You-chickens achieved by genome resequencing remains limited. Therefore, by using the data from two fast-growing European-origin broiler lines (BRs), including broiler line A (BRA) and broiler line B (BRB), as reference populations, the genetic structure and selected regions of BCY, BJY, and BRs were analyzed, thus providing a deeper understanding of the germplasm resources and the basis of molecular breeding of Chinese-You chickens.

## 2. Materials and Methods

### 2.1. Birds

Genome resequencing data from 103 individuals were used in the present study. In particular, we sequenced 31 BCY chickens (16 males and 15 females) from the BCY Chicken Conservation Farm (Baicheng, Xinjiang, China) and 32 BJY chickens (16 males and 16 females) from the BJY Chicken Conservation Farm, Beijing Academy of Agriculture and Forestry Sciences (Beijing, China). The BCY population was established in 2007 and maintained at a population size of more than 3000 birds. The BJY population was developed in 1972; the size of the conservation population varies between 3000 and 4000 birds in each generation. All animal handling and collection procedures were approved by the Animal Ethics Committee of the Nanjing Agricultural University (No. 20220318053). Additionally, resequencing data, including that of 20 BRA and 20 BRB chickens originating from Germany and France, respectively, were downloaded from the European Nucleotide Archive (No. PRJEB30270), which was produced in a previous study [5].

### 2.2. Library Construction and Sequencing

DNA was extracted from the blood of BCY and BJY chickens using the phenol-chloroform extraction method. DNA purity, integrity, and quantification were detected using a NanoDrop 2000 (ThermoFisher Scientific, San Jose, CA, USA), an agarose electrophoresis, and a Qubit Fluorometer (ThermoFisher Scientific, San Jose, CA, USA), respectively. Libraries for sequencing were set up using a DNA library construction kit (Illumina, San Diego, CA, USA) and sequenced using the Illumina NovaSeq 6000 sequencing platform (Berry Genomics, Beijing, China).

### 2.3. Genome Mapping, SNP Calling, and Filtering

After removing reads with an adaptor or N ratio > 10% and low-quality reads, we mapped the remaining clean reads to the chicken reference genome (Gallus gallus_GRCg6a) using the Burrows–Wheeler Aligner v0.7.15 [9] with the parameter “bwa mem -t 8 -M -R” and discarded the duplicate reads by PICARD 1.57 (github.com/broadinstitute/picard) with the parameter “picard MarkDuplicates”. SNPs were developed using GATK 4.0.0.0 [10] with the following parameters: “QD < 2.0”, “FS > 200.0”, “SOR > 10.0”, “MQRankSum < −12.5”, and “ReadPosRankSum < −8.0”. Filtering of high-quality SNPs was performed using PLINK v1.90 [11]. The Hardy–Weinberg equilibrium (HWE) test for autosomal SNPs was performed using PEDSTATS 0.6.12 [12].

### 2.4. Population Genetic Analysis

The population structure was analyzed using ADMIXTURE 1.3 [13], and four independent runs were performed with K ranging from 2 to 5 and then plotted using RStudio. Gene diversity within the population was estimated using the R package of adegenet with the “df2genind” function, while observed heterozygosity and IBS genetic distance were calculated using PLINK v1.90 with the commands as “--het” and “--cluster --matrix --noweb”, respectively. The phylogenetic tree was drawn using MEGA 7.0 [14]. The pairwise genetic differentiation coefficient (*F*_ST_) values between the two populations were computed using VCFTOOLS 0.1.15 [15] with the command “--weir-fst-pop”.

### 2.5. Selected Region Analysis

The selected regions were determined using the *F*_ST_ and cross-population extended haplotype homozygosity (XP-EHH) methods as described in previous studies with slight modifications: (1) *F*_ST_ values of each single nucleotide polymorphism (SNP) were calculated using R 4.1.1 with the previous algorithm [16,17], while VCF files were phased using Beagle 4.1 and then used to calculate the XP-EHH values of each SNP using SELSCAN 1.3.0 [18,19]. (2) For the *F*_ST_ method, the Fisher’s exact test and Bonferroni correction were applied to calculate the difference in allele frequencies between BJY and BRs chickens and between BCY and BJY chickens, respectively. (3) SNPs with the 0.01% highest *F*_ST_ values and corrected *p* values < 0.01 or 0.1% highest |XP-EHH| values were regarded as extremely significant SNPs. (4) SNPs with *F*_ST_ values ≥ 0.4 and corrected *p* < 0.01 or |XP-EHH| values ≥ 1 were considered as significant SNPs. (5) Centering on the extremely significant SNPs, neighboring SNPs were used to define the boundaries of the region until more than two consecutive non-significant SNPs were encountered. When a region had more than five significant SNPs but no extremely significant SNPs, it was also regarded as a selected region.

The cross-population composite likelihood ratio (XP-CLR) method was also used to identify the selected region using the XPCLR program [20]. The parameters were set as 25 kb length for non-overlapping sliding windows, a maximum of 500 SNPs for each window, and 0.95 for linkage disequilibrium cutoff to apply for weighting. A region with the top 1% of xpclr_norm values was defined as a candidate region.

Genes from the above-selected regions were identified using the annotation of the chicken reference genome (Gallus gallus_GRCg6a.104).

## 3. Results

### 3.1. Characteristics of Sequencing Data

A total of 343 GB of data from 63 Chinese-You chickens was produced by genome resequencing. As shown in Table S1, an average of 30,686,143 clean reads was obtained after quality control. The average Q30, GC content, and cover depth were 94.48%, 42.82%, and 8.20×, respectively. Moreover, an average mapping rate of 98.54% was obtained using the chicken reference genome.

### 3.2. Identification of SNPs in BCY, BJY, and BRs Chickens

Combined with the sequencing data from 20 BRA and 20 BRB individuals, 18,852,372 SNPs were discovered in 103 individuals. After SNP filtering (Table S2), 8,207,242 autosomal SNPs were included in the selected region analysis. Meanwhile, a subset containing 5,158,483 autosomal SNPs with MAF ≥ 0.2 was applied for genetic diversity analysis.

### 3.3. Accessing the Genetic Diversity

**Figure 1:**
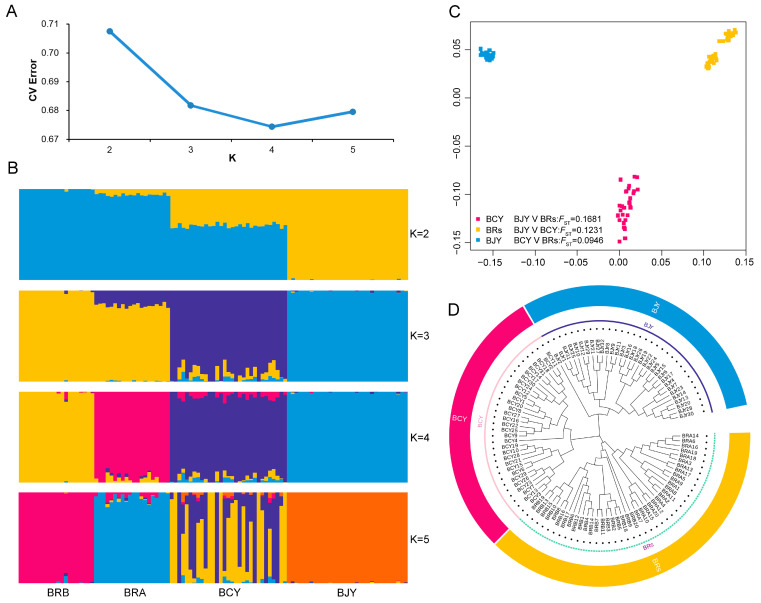
Population structure analysis. (**A**,**B**) Population structure of European-origin broiler lines (BRs), Baicheng-You (BCY), and Beijing-You (BJY) chickens revealed by ADMIXTURE analysis. (**C**) Multidimensional scaling plots among BCY, BJY, and BRs chickens using the PLINK v1.90 software. (**D**) Phylogenetic tree among BCY, BJY, and BRs chickens.

Population structure analysis showed that BRA, BRB, BCY, and BJY chickens formed four independent populations (*K* = 4) (Figure 1A,B). Moreover, BRA and BRB formed one cluster in the phylogenetic tree (Figure 1D). Since BRA and BRB originated in Europe, they were merged as one population (BRs) and compared with the two Chinese You-chickens in the subsequent analysis. The results from the pairwise *F*_ST_ indicated a relatively large genetic differentiation between BJY and BRs (*F*_ST_ = 0.1681) and between BCY and BJY (*F*_ST_ = 0.1231), whereas there was a closer genetic distance between BCY and BRs (*F*_ST_ = 0.0946) (Figure 1C). Furthermore, the observed heterozygosity of BCY, BJY, and BRs was 0.3500, 0.3457, and 0.3566, respectively, whereas the gene diversity of BCY, BJY, and BRS was 0.5513, 0.5060, and 0.5351, respectively.

### 3.4. Selected Region Analysis between BJY and BRs Chickens

**Figure 2:**
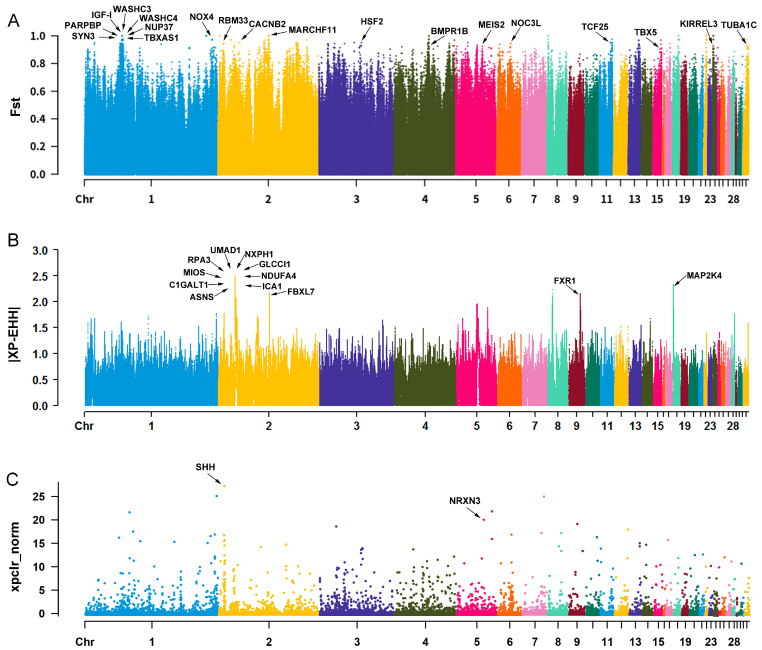
Selected region analysis between Beijing-You (BJY) and European-origin broiler lines (BRs). Manhattan plot of genetic differentiation coefficient (*F*_ST_) values (**A**), cross-population extended haplotype homozygosity (XP-EHH) values (**B**), and cross-population composite likelihood ratio (XP-CLR) scores (xpclr_norm) (**C**) of BJY chickens compared to BRs chickens. The genes marked by the arrows contained SNPs with extremely high *F*_ST_ or |XP-EHH| values or were located in the regions with extremely high XP-CLR scores. The threshold of extremely high *F*_ST_, |XP-EHH|, and xpclr_norm score was greater than 0.9, 2, and 20, respectively.

A total of 11,407 selected regions, including 1779 unique genes from the *F*_ST_ method, 1600 selected regions, including 428 unique genes from the XP-EHH test, and 384 selected regions, including 243 unique genes from XP-CLR analysis (Tables S3–S5), were identified between BJY and BRs chickens. Among these, 374 genes discovered by at least two of the three methods were defined as the final candidate genes (Table S6). Moreover, we identified many candidate genes associated with important economic traits such as *INSR*, *ELOVL5*, *LCAT*, and *NOC3L* (Table 1). Additionally, some candidate genes also contained SNPs with extremely high *F*_ST_ or XP-EHH values or were located in regions with extremely high XP-CLR scores, such as *IGF-I*, *FXR1*, *NOX4*, *ASNS*, *UMAD1*, *GLCCI1*, and *ICA1* (Figure 2A–C).

### 3.5. Selected Region Analysis between BCY and BJY Chickens

**Figure 3:**
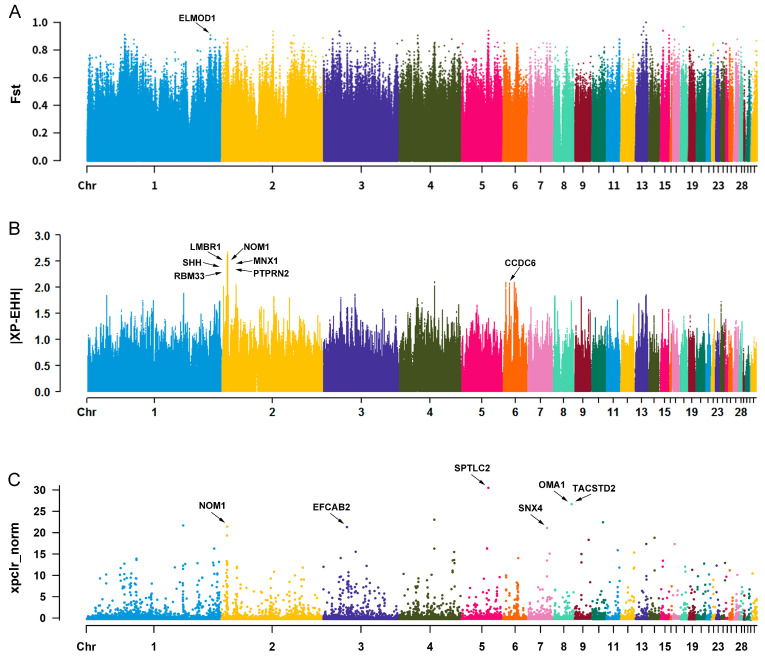
Selected region analysis between Baicheng-You (BJY) and Beijing-You (BCY) chickens. Manhattan plot of genetic differentiation coefficient (*F*_ST_) values (**A**), cross-population extended haplotype homozygosity (XP-EHH) values (**B**), and cross-population composite likelihood ratio (XP-CLR) scores (xpclr_norm) (**C**) of BJY compared to BCY chickens. The genes marked by the arrows contained SNPs with extremely high *F*_ST_ or |XP-EHH| values or were located in the regions with extremely high XP-CLR scores. The threshold of extremely high *F*_ST_, |XP-EHH|, and xpclr_norm score was greater than 0.9, 2, and 20, respectively.

A total of 5141 selected regions, including 926 unique genes from the *F*_ST_ method, 2382 selected regions, including 499 unique genes from the XP-EHH test, and 384 selected regions, including 219 unique genes from the XP-CLR analysis (Tables S7–S9), were identified between BCY and BJY chickens. A total of 279 genes were identified as final candidate genes with at least two of the three methods (Table S10). Among them, many candidate genes associated with important economic traits were identified, including *ASIP*, *PAH*, *IL18*, *PLXNA4*, and *ESRRG* (Table 2). Additionally, some candidate genes also contained SNPs with extremely high *F*_ST_ or XP-EHH values or were located in regions with extremely high XP-CLR scores, such as *ELMOD1*, *CCDC6*, *SPTLC2*, and *OMA1* (Figure 3A–C).

### 3.6. Identification of Selected Genes in BJY Chickens

After the identification of 374 and 279 selected genes between BJY and BRs chickens and between BCY and BJY chickens, respectively, we observed that 104 genes overlapped (Table S11), including *DNAJB6*, *WNT11*, *WNT9A*, *SHH*, and *LMBR*. These genes may represent the unique genomic characteristics of BJY chickens, distinguishing them from BCY and BRs chickens.

## 4. Discussion

In the present study, we found that BJY and BRs chickens have the least genetic relationship, whereas BCY and BRs chickens have a greater genetic relationship (Figure 1C). This is not surprising because the other chicken breeds, including Tulufan and Hetian chickens, originating from Xinjiang, and European-origin chickens, were clustered in the same clade, whereas the chicken breeds originating from Beijing belonged to a different clade [56], which is similar to our results. From a genome-wide perspective, this is the first report that BCY and BJY chickens have a distant genetic relationship compared to European-origin chickens, although they are all called “You-chicken” in China. Among these chicken breeds, the results of observed heterozygosity and gene diversity analyses indicated that BJY chickens had lower genetic diversity. Overall, our results provide insight into the genetic structure and genetic relationship between two European-origin chicken lines and two Chinese You-origin chicken breeds.

BJY chicken is a slow-growing broiler breed with excessive fat deposition, whereas BRs chickens are fast-growing broiler strains. As expected, several genes related to muscle growth and fat deposition, such as *IGF-I*, *INSR*, *ELOVL5*, *LCAT*, and *NOC3L*, were identified between BJY and BRs chickens (Table 1). As a key gene related to body growth and development, *IGF-I* can promote the growth of lean meat and feed efficiency of broilers [57], and its polymorphisms are correlated with body mass, breast muscle weight, and abdominal fat deposition in different broiler lines [21,58]. INSR, a cognate receptor of IGF-I, was found to be involved in fat deposition in Ethiopian indigenous sheep [39]. Consequently, our study provides further evidence for the function of *IGF-I* and *INSR* in the growth of broilers. ELOVL5 is a fatty acid elongase, and its mutations were found to affect subcutaneous fat thickness in cattle [59]. In chickens, the expression of the miR-10a-5p-*ELOVL5* pair has been associated with abdominal adipocyte differentiation [29]. LCAT is considered a new adipokine that has higher expression levels in visceral fat tissues in broilers than in layers [36], whereas NOC3L is considered an influencing factor for promoting adipogenesis in mice [32]. These studies confirmed that *ELOVL5*, *LCAT*, and *NOC3L* have important functions in adipogenesis. Thus, our data may indicate a new clue for their function in chicken fat deposition. In addition, we found that some genes with extremely high *F*_ST_ or X-PEHH values are involved in other economic traits. For example, *FXR1*, *ASNS*, and *NOX4* affect heat stress [23,24,34], and *UMAD1*, *GLCCI1*, and *ICA1* affect eggshell color (Table 1 and Figure 2A,B) [25]. Modern broilers are increasingly vulnerable to heat stress because of their excessive growth rate. Mutations in *FXR1*, *ASNS*, and *NOX4* might result in different responses of BJY and BRs chickens to thermal stress, but this hypothesis needs further verification. Recently, using the same BRA and BRB broiler populations, another study found that *UMAD1*, *GLCCI1*, and *ICA1* are located at eggshell color-related QTLs [25]. The eggshell color of BJY chickens is pink or light pink [1], whereas that of BRs chickens is brown or light brown (https://aviandiv.fli.de/, accessed on 1 January 2022), implying that there are differences in eggshell color between BJY and BRs chickens. Our data also suggest that these genes may be good candidates for eggshell color traits. However, further investigations are needed to elucidate the mechanism by which *UMAD1*, *GLCCI1*, and *ICA1* affect eggshell color in chickens.

As described above, BCY and BJY are the only two kinds of You-chickens in China, but there is a distant genetic relationship between them. BCY chickens mainly have black and brown feathers, whereas BJY chickens have yellow feathers. In this study, two important genes involved in melanin formation, *ASIP* and *PAH*, were observed in the selected regions of these two chicken breeds (Table 2). As a well-known regulator of melanin synthesis, the expression and polymorphisms of *ASIP* have been shown to affect feather color in chickens [54]. *PAH* is a phenylalanine hydroxylase that converts phenylalanine to tyrosine, a rate-limiting substrate for melanin formation. In silkworms, *PAH* is necessary for melanin biosynthesis [41]. However, its role in chicken remains unclear. Our data provide a new concept that explains the action of *ASIP* and *PAH* on melanin formation in chickens. Interestingly, many genes involved in adaptability to harsh environments were also identified in the selected regions. The annual average temperature and precipitation in Baicheng are 7.6 °C and 171 mm, respectively, whereas the annual average temperature and precipitation in Beijing are 11 °C and 644 mm, respectively [1], indicating that the climate in Baicheng is colder and drier than that in Beijing. As a result, BCY chickens may have stronger immunity to adapt to harsh environments. We discovered that *IL18*, *PLXNA4*, *SPTLC2*, and *MARCH1* are involved in immune responses (Table 2). As a cytokine of the IL-1 family, *IL18* has been shown to increase immune responses in multiple chicken diseases [60] and is regarded as an adaptive gene for tropical and harsh environments in chickens [61,62], which is similar to our data. Moreover, *PLXNA4* and *SPTLC2* are regulators of T cell-mediated immune responses and protective immunity [40,51], whereas *MARCH1* is a multifunctional regulator of adaptive immunity [50]. Our results demonstrated that these genes could function in chicken immune responses. Furthermore, extremely high *F*_ST_ values were observed for *ELMOD1* (Table 2 and Figure 3A), a gene involved in the development of hair cells [63]. Mutations in *ELMOD1* caused deafness and hair cell dysfunction in mice [64]. However, to date, the role of *ELMOD1* in chickens has not been reported. Overall, our research provides a first-hand reference for understanding the genomic differences between these two Chinese You chickens.

Additionally, we found that some important genes related to feather growth were selected in BJY chickens (Table S11), including *SHH*, *WNT9A*, and *WNT11*. As a key gene in hair follicle development in feathers, *SHH* is expressed during feather formation in chickens [65] and mainly affects mitosis and morphogenesis during the development of feather buds [66]. Silencing and overexpression of *SHH* resulted in irregular and enlarged feather buds during feather development in chickens, respectively [67,68]. *WNT9A* affects feather growth in birds [69], whereas *WNT11* influences the size and shape of feather buds, and indels in this gene are related to the feathered-leg character of chickens [70,71]. These studies showed that *SHH*, *WNT9A*, and *WNT11* may play important roles in feather growth. Compared to BCY and BRs chickens, BJY chickens have unique feather growth characteristics, including tibial feathers, toe feathers, and beards [1]. Accordingly, our data might provide new evidence for the role of *SHH*, *WNT9A*, and *WNT11* in feather growth in BJY chickens. More interestingly, the three nearest neighbors to *SHH*, including *NOM1*, *LMBR1*, and *RBM33*, were identified as selection genes in BJY chickens compared to BCY and BRs chickens (Table S11). These genes belong to the long-range regulatory domain of *SHH* [72]. Previously, mutations in *LMBR1* were associated with polydactyly in multiple chicken populations, including BJY chickens [73,74]. However, the functions of *NOM1* and *RBM33* in agricultural animals have not been clarified. Our results imply that these genes might be related to the unique characteristics of BJY chickens; however, confirmation of this assumption requires further study.

## 5. Conclusions

In summary, this study revealed the genetic relationships among BRs, BCY, and BJY chickens. Moreover, selected regions containing a large number of functional genes related to specific breed characteristics were also discovered, such as *SHH*, *WNT9A*, and *WNT11* for feather growth and *ELOVL5*, *LCAT*, and *NOC3L* for fat deposition in BJY chickens, *IL18*, *PLXNA4*, *SPTLC2*, and *MARCH1* for harsh environmental adaptability in BCY chickens, as well as *IGF-I* for growth and *UMAD1*, *GLCCI1*, *ICA1* for eggshell color in BRs chickens. These results provide valuable clues for elucidating the formation of fat deposition and potential disease resistance in the broilers, which can be used to improve the production performance of local chicken breeds while preserving their appearance features in subsequent breeding.

## Figures and Tables

**Table 1:** Candidate genes associated with important functions between Beijing-You and European-origin broiler lines.

Chr	Position		Gene	Methods	Function
1	55281097	55330373	*IGF-I*	*F*_ST_, XP-CLR	Fat content in chickens [21]
1	65833774	66173568	*SOX5*	*F*_ST_, XP-EHH	Abdominal fat deposition in chickens [22]
1	189025780	189161699	*NOX4*	*F*_ST_, XP-EHH	Heat stress in chickens [23]
2	24662430	24676154	*ASNS*	*F*_ST_, XP-EHH	Heat shock in mice [24]
2	24794127	24862531	*UMAD1*	*F*_ST_, XP-EHH	Eggshell color in chickens [25]
2	24891742	24943662	*GLCCI1*	*F*_ST_, XP-EHH	Eggshell color in chickens [25]
2	24954107	25024569	*ICA1*	*F*_ST_, XP-EHH	Eggshell color in chickens [25]
2	80852532	80991947	*GRB10*	*F*_ST_, XP-EHH	Lipolysis and thermogenesis in mice [26]
2	116504849	116596399	*NCOA2*	*F*_ST_, XP-EHH	Preadipocyte differentiation in humans [27]
3	29410438	29492850	*GLP1R*	*F*_ST_, XP-EHH	Chicken abdominal fat [28]
3	88246591	88283573	*ELOVL5*	*F*_ST_, XP-EHH	Adipocyte differentiation in chickens [29]
4	82843121	82879154	*MXD4*	*F*_ST_, XP-EHH	Novel regulators of adipogenesis in humans [30]
5	30776671	30777369	*GREM1*	*F*_ST_, XP-EHH	Novel adipokines in humans [31]
6	21058455	21075606	*NOC3L*	*F*_ST_, XP-EHH	Hyperplasia in adipose tissue in mice [32]
8	7250615	7378011	*COP1*	*F*_ST_, XP-EHH	Fat metabolism in mice [33]
9	17247026	17278252	*FXR1*	*F*_ST_, XP-EHH	Environmental stress in humans [34]
9	17441931	17531426	*PEX5L*	*F*_ST_, XP-EHH	Chicken adipogenesis in liver [35]
11	1034930	1041693	*LCAT*	XP-EHH, XP-CLR	New adipokines in chickens [36]
15	9670176	9678598	*SIRT4*	*F*_ST_, XP-CLR	Adipose pathology in mice [37]
15	12598201	12638563	*TBX5*	*F*_ST_, XP-CLR	Feathered legs in chickens [38]
28	4218437	4253117	*INSR*	*F*_ST_, XP-EHH	Fat deposition in sheep [39]

Note: *IGF-I*: insulin like growth factor 1; *SOX5*: SRY-box 5; *NOX4*: NADPH oxidase 4; *ASNS*: asparagine synthetase; *UMAD1*: UBAP1-MVB12-associated (UMA) domain containing 1; *GLCCI1*: glucocorticoid induced 1; *ICA1*: islet cell autoantigen 1; *GRB10*: growth factor receptor bound protein 10; *NCOA2*: nuclear receptor coactivator 2; *GLP1R*: glucagon like peptide 1 receptor; *ELOVL5*: ELOVL fatty acid elongase 5; *MXD4*: MAX dimerization protein 4; *GREM1*: gremlin 1, DAN family BMP antagonist; *NOC3L*: NOC3 like DNA replication regulator; *COP1*: COP1 E3 ubiquitin ligase; *FXR1*: FMR1 autosomal homolog 1; *PEX5L*: peroxisomal biogenesis factor 5 like; *LCAT*: lecithin-cholesterol acyltransferase; *SIRT4*: sirtuin 4; *TBX5*: T-box 5; *INSR*: insulin receptor.

**Table 2:** Candidate genes associated with important functions between Baicheng-You and Beijing-You chickens.

Chr	Position		Gene	Methods	Function
1	2887148	3334587	*PLXNA4*	*F*_ST_, XP-EHH	Immune responses in mice [40]
1	55078825	55114899	*PAH*	*F*_ST_, XP-EHH	Melanin biosynthetic in bombyx mori [41]
1	77477877	77514773	*KPNA1*	*F*_ST_, XP-EHH	Melanogenic process in guinea pigs [42]
1	141624410	141643190	*TNFSF13B*	*F*_ST_, XP-CLR	Immune suppression in chickens [43]
1	195484990	195565945	*UVRAG*	*F*_ST_, XP-EHH	Skin innate immunity in mammals [44]
2	2551903	2634749	*WNT3A*	*F*_ST_, XP-EHH	Melanin synthesis in mice [45]
2	21172333	21382361	*ZNF804B*	*F*_ST_, XP-EHH	Skin and iris color in humans [46]
2	22703461	22836675	*CDK6*	*F*_ST_, XP-EHH	Immunotherapies in humans [47]
3	20068343	20429542	*ESRRG*	*F*_ST_, XP-EHH	Mitochondrial thermogenesis in mice [48]
3	34048590	34412338	*SMYD3*	*F*_ST_, XP-EHH	Immune system in humans [49]
4	23437223	23636944	*MARCH1*	*F*_ST_, XP-EHH	Immunomodulation in humans [50]
5	39473855	39531939	*SPTLC2*	XP-EHH, XP-CLR	Protective immunity in humans [51]
8	26650130	26666710	*OMA1*	*F*_ST_, XP-EHH, XP-CLR	Mitochondrial thermogenesis in mice [52]
16	2609635	2624278	*C4*	*F*_ST_, XP-EHH	Autoimmunity in humans [53]
20	1567219	1596889	*ASIP*	XP-EHH, XP-CLR	Chicken plumage color [54]
24	6169486	6173722	*IL18*	XP-EHH, XP-CLR	Immune regulation in humans [55]

Note: *PLXNA4*: plexin A4; *PAH*: phenylalanine hydroxylase; *KPNA1*: karyopherin subunit alpha 1; *TNFSF13B*: tumor necrosis factor superfamily member 13b; *UVRAG*: UV radiation resistance-associated; *WNT3A*: Wnt family member 3A; *ZNF804B*: zinc finger protein 804B; *CDK6*: cyclin-dependent kinase 6; *ESRRG*: estrogen-related receptor gamma; *SMYD3*: SET and MYND domain containing 3; *MARCH1*: membrane-associated ring-CH-type finger 1; *SPTLC2*: serine palmitoyltransferase long chain base subunit 2; *OMA1*: OMA1 zinc metallopeptidase; *C4*: complement 4; *ASIP*: agouti signaling protein; *IL18*: interleukin 18.

## Data Availability

Genome resequencing datasets: 31 BCY chickens and 32 BJY chickens are available from the SRA database (accession number PRJNAT792508).

## Supplementary Materials

The following supporting information can be downloaded at https://www.mdpi.com/article/10.3390/biology12111397/s1, Table S1: Characteristics of sequencing data in Baicheng-You and Beijing-You chickens; Table S2: Quality-control for filtering SNPs. Table S3: Candidate genes between Beijing-You and European-origin broilers (BRs) identified by the *F*_ST_ method; Table S4: Candidate genes between Beijing-You and European-origin broilers (BRs) identified by the XP-EHH method; Table S5: Candidate genes between Beijing-You and European-origin broilers (BRs) identified by the XP-CLR method; Table S6: Overlapping genes between Beijing-You and European-origin broilers (BRs) identified using at least two methods; Table S7: Candidate genes between Baicheng-You and Beijing-You chickens identified by the *F*_ST_ method; Table S8: Candidate genes between Baicheng-You and Beijing-You chickens identified by the XP-EHH method; Table S9: Candidate genes between Baicheng-You and Beijing-You chickens identified by the XP-CLR method; Table S10: Overlapping genes between Baicheng-You and Beijing-You chickens identified using at least two methods; Table S11: The selected genes in Beijing-You chickens compared with Baicheng-You and European-origin broilers (BRs).
